# Spin chemistry in living systems

**DOI:** 10.1093/nsr/nwae126

**Published:** 2024-03-28

**Authors:** P J Hore

**Affiliations:** Department of Chemistry, Oxford University, UK

Magnetobiology—the study of non-thermal effects of magnetic fields on biological systems—has a vast, wide-ranging and ever-expanding literature. Sadly, much of it is beset by conflicting, implausible or extravagant claims. The credibility of many reports is marred by shortcomings in experimental design and description, inadequate controls, unblinded protocols, inappropriate statistical analysis, lack of comparability with other studies or poor reproducibility. Careful attempts at direct independent replication are scarce and frequently unsuccessful [1,2]. Authors can be reluctant to write up, journal editors to publish, and others to cite negative replication studies, resulting in an unbalanced literature. False positives no doubt abound. Raw data are often not made publicly available. The magnetobiology literature must therefore be viewed with a critical eye: not everything should be accepted at face value. This is a shame. Magnetic field effects on *chemical* systems have provided powerful insights into the structures and dynamics of molecules and the kinetics and mechanisms of their reactions [3,4]. Related achievements should be possible in biology, in areas such as the physiological effects of anthropogenic magnetic fields, the effectiveness of magnetic therapies and medical diagnostics, and the improvement of crop yields, to name but a few.

A major obstacle in many of these investigations is the shortage of established molecular interaction mechanisms that can be used to design experiments and interpret data. By far the most promising is the radical pair mechanism (RPM), a central part of spin chemistry. The RPM offers a framework for understanding and analysing the (bio)chemical consequences of magnetic interactions whose energies are dwarfed by the ever-present chaotic thermal motions of molecules [5]. In laboratory studies over the last 60 years, hundreds of chemical transformations have been shown to be consistent with RPM theory. Could the RPM also be important in living systems? Opinions vary from enthusiastic cheerleading to hard-nosed scepticism. If the RPM is genuinely relevant in biology, then it has the potential to separate some of the wheat from the chaff in the magnetobiology literature (Supplementary Material).

The application of the RPM that has attracted the most attention in recent years is the magnetic compass sense of migratory songbirds. Birds use the Earth's magnetic field (∼50 μT) as a navigational cue, and the evidence suggests that the primary sensors are cryptochrome flavoproteins located in photoreceptor cells in the birds’ eyes (Fig. 1). Although many details are far from clear, it appears that light-induced intra-protein electron transfer reactions produce a magnetically sensitive flavin-tryptophan radical pair and hence a signalling state whose quantum yield encodes the direction of the magnetic field (Supplementary Material) [6]. Currently the most persuasive evidence for this hypothesis comes from a specialized behavioural experiment that could be described as ‘animal-detected magnetic resonance’. It is clear, both experimentally and theoretically, that the response of a radical pair reaction to a *static* magnetic field can be modified by a weak *time-dependent* magnetic field if it oscillates at one of the frequencies with which the singlet and triplet radical pairs coherently interconvert. For flavin-tryptophan pairs, such resonances are predicted to occur for radiofrequencies up to but not beyond ∼116 MHz. Remarkably, Eurasian blackcaps subject to weak noise-modulated radiofrequency fields (∼3 ${\mathrm{pT}}/\sqrt {{\mathrm{Hz}}} $) are able to use their magnetic compass at frequencies above, but not below, ∼116 MHz, suggesting that resonant radiofrequency fields interfere with the spin dynamics of the radical pairs and corrupt the directional information they provide [7].

**Figure 1.**
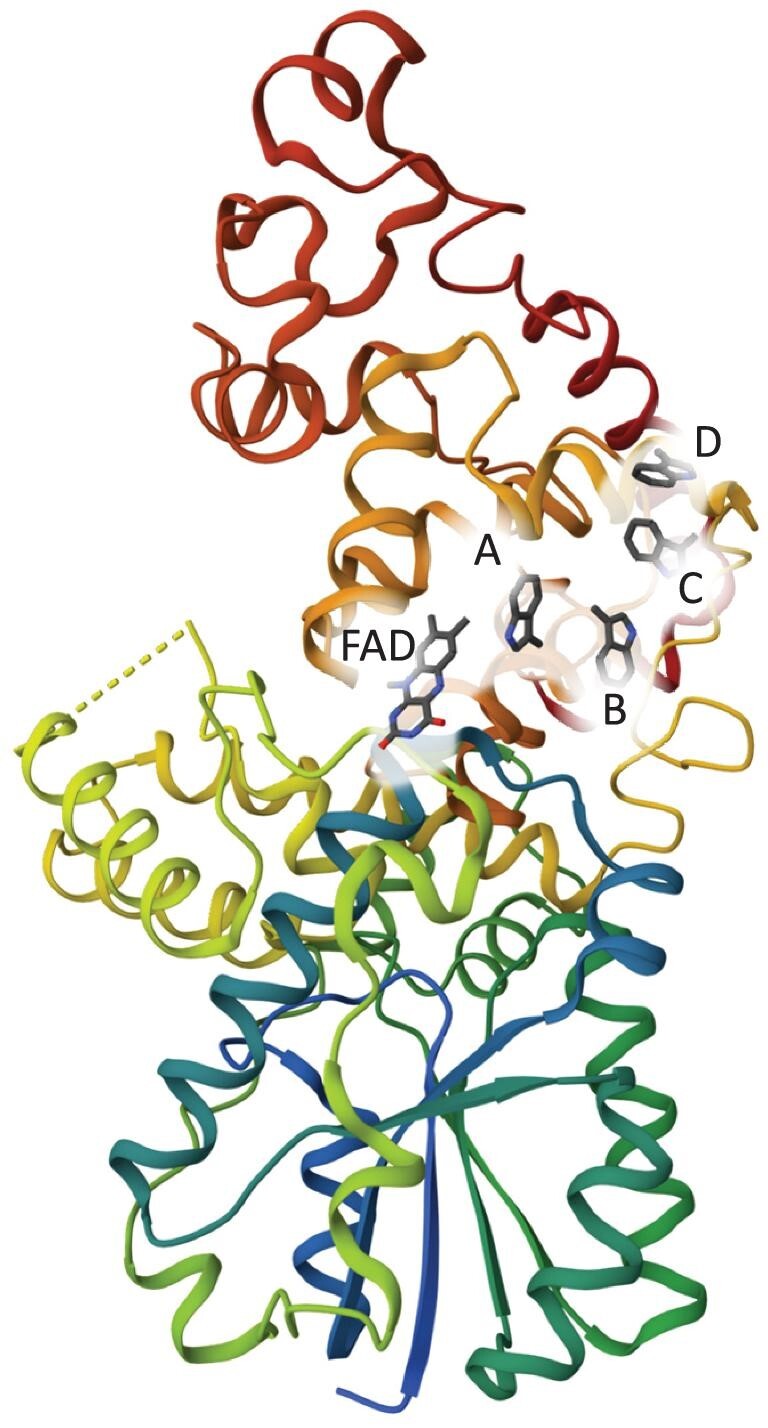
Cryptochrome-4a has been proposed as the receptor for the magnetic compass sense of migratory songbirds. Radical pairs are formed by blue-light excitation of the flavin group of the FAD (flavin adenine dinucleotide) chromophore followed by sequential electron transfers along a chain of four tryptophan residues (A–D).

However, there is still much to be done to convince sceptics that the RPM is at the heart of the avian magnetic compass [5]. Reproducible changes in the photochemistry of purified cryptochromes have yet to be detected for magnetic fields weaker than ∼1 mT and, so far, there have been no reports of the anisotropic magnetic responses that would be required of a direction sensor. It is uncertain which of the six avian cryptochromes might be involved, although Cry4a seems by far the most likely. Nor is it clear that flavin-tryptophan is the correct radical pair. Some authors favour a pair in which the tryptophan is replaced by superoxide, ${\mathrm{O}}_2^{ \bullet - }$, or some unknown radical. Although circumstantial evidence favours cryptochrome, it is possible that we may be barking up the wrong tree [8]. What is crucially needed is a definitive experiment with the power to prove or disprove the existence of a cryptochrome-based radical pair magnetoreceptor in the eyes of migratory songbirds.

The burgeoning popularity of the cryptochrome hypothesis (Supplementary Material) has spawned an enthusiasm for the RPM in other areas of magnetobiology, in particular those that are known to involve, or might conceivably involve, flavins, flavoproteins, tryptophan or superoxide [9]. Flavin-superoxide radical pairs, in particular, have attracted attention because of the importance of reactive oxygen species (ROS, e.g. ${\mathrm{O}}_2^{ \bullet - }$, ${\mathrm{O}}{{{\mathrm{H}}}^ \bullet }$ and ${\mathrm{N}}{{{\mathrm{O}}}^ \bullet }$) in cell signalling, oxidative damage and (perhaps) magnetic therapies. Various ROS-related magnetic field effects have been reported for static, time-dependent and hypomagnetic (i.e. <<50 μT) fields. Superoxide also seems to be a favourite amongst theoreticians: the absence of internal magnetic interactions (^16^O, the dominant oxygen isotope, has no nuclear magnetic moment) seems to make ${\mathrm{O}}_2^{ \bullet - }$ an irresistible candidate for quantum spin dynamics simulations. Nevertheless, there is clear experimental evidence and persuasive theoretical arguments that ${\mathrm{O}}_2^{ \bullet - }$ spin-relaxes so rapidly (∼nanoseconds) that any effects of weak magnetic fields should be entirely negligible. If independently reproducible RPM effects on ROS levels in living systems do exist, then the ROS are much more likely to be produced downstream of a radical pair comprising slower relaxing organic radicals.

There have also been reports of cryptochrome-mediated magnetic field effects on plant cells and intact plants, many of which have been attributed to the RPM [10]. Plants, unlike long-distance migrants, presumably have no use for a magnetic compass and may derive some other evolutionary benefit from the presence of the geomagnetic field. Optimization of growth, for example, seems more likely than the development of a new sensory modality.

The magnetic (hyperfine) coupling of electronic and nuclear spins plays a major role in determining the magnetic sensitivity of radical pair reactions. A natural consequence is that isotopic substitution can also alter reaction rates and yields if the nuclides have different magnetic moments. Such effects are well-established for chemical systems and have been the focus of some intriguing work on biological catalysis. Magnesium magnetic isotope effects have been reported for two important classes of enzymatic reactions both of which require Mg^2+^ ions and involve formation of covalent phosphorus-oxygen bonds, namely conversion of ADP to ATP and DNA synthesis [11]. Similar effects have been claimed for isotopes of Ca^2+^ and Zn^2+^. In contrast to the conventional mechanism (nucleophilic substitution), it has been proposed that these reactions involve a radical pair comprising a phosphate radical and a singly charged metal ion ${{{\mathrm{M}}}^{ \bullet + }}$(M = Mg, Ca, Zn) (Supplementary Material). There appears to be no previous suggestion of a radical mechanism or, indeed, any redox biochemistry of these three elements. Aside from the exotic nature of the metal radicals, the proposed mechanism appears to account qualitatively for most of the reported effects. Given the importance of DNA replication, transcription and repair, it is odd that this work has attracted so little interest (with the exception of one negative replication study). There have also been attempts to rationalize isotope effects on anaesthesia, hyperactivity and circadian timing via radical pairs in which one radical is ${\mathrm{O}}_2^{ \bullet - }$ (see above) complexed to either a Xe atom or a Li^+^ ion [12].

One of the beauties of the RPM is that it offers a sound theoretical framework for interpreting data and making predictions that can be tested experimentally. Agreement between theory and experiment is clearly desirable but to be meaningful, simulations should use sufficiently realistic models despite the computational burden. Care is needed: over-simplified models can easily be misleading, both qualitatively and quantitatively. Computationally inconvenient but important factors such as spin relaxation and exchange/dipolar couplings should not be swept under the carpet. The more realistic the simulations the smaller the predicted effects often become.

In practice, multiple constraints (chemical, magnetic, kinetic, structural etc.) need to be satisfied for a radical pair reaction to show measurable responses to weak magnetic fields [13]. Unsurprisingly, the weaker the field, the more stringent the criteria. Very few convincing RPM effects have been reported for *chemical* systems subject to magnetic fields weaker than ∼100 μT even in studies where the experimental conditions can be optimized in a variety of ways (temperature, pH, ionic strength, concentration, solvent, wavelength and intensity of light and so on). Radical pair reactions in cells and whole organisms, with efficient homeostatic, defence and repair mechanisms, offer fewer opportunities for experimental optimization and are therefore arguably even less likely to show pronounced effects of <100 μT fields unless they have been subject to evolutionary pressure and/or have efficient signal transduction and amplification mechanisms.

Changes in the yield of a radical pair reaction induced by the 50/60 Hz magnetic fields from electrical appliances and power transmission lines to which humans are routinely exposed have been estimated at no more than 10 parts per million [14]. To put this in context, a similar change in reaction yield would be expected from a 2–10 nT change in the geomagnetic field, such as would be experienced by travelling a few kilometres towards or away from the geomagnetic north or south pole, or from the natural diurnal variation in the Earth's field (25–50 nT).

Spin chemistry is more than just the RPM. Other mechanisms—less common in chemical systems but arguably relevant in biology—involve molecular triplet states, radical scavenging (which may circumvent the problems surrounding ${\mathrm{O}}_2^{ \bullet - }$), radical triads and the intriguing phenomenon of chirality-induced spin selectivity [15]. Moreover, there are spin chemical effects that probably have no biological consequences but which are of great value in understanding cellular processes. An example is the electron and nuclear spin hyperpolarization formed during the initial steps of photosynthetic energy conversion, which has provided much information on the intricacies of light-induced charge separation and stabilization.

## Supplementary Material

nwae126_Supplemental_File

## References

[1] Bassetto M, Reichl T, Kobylkov D et al. Nature 2023; 620: 595–9. 10.1038/s41586-023-06397-7 37558871 PMC10432270

[2] Uzhytchak M, Smolkova B, Frtus A et al. Sci Rep 2023; 13: 10818. 10.1038/s41598-023-38015-x 37402779 PMC10319795

[3] Mims D, Herpich J, Lukzen NN et al. Science 2021; 374: 1470–4. 10.1126/science.abl4254 34914495

[4] Harvey SM, Wasielewski MR. J Am Chem Soc 2021; 143: 15508–29. 10.1021/jacs.1c07706 34533930

[5] Hore PJ, Mouritsen H. Annu Rev Biophys 2016; 45: 299–344. 10.1146/annurev-biophys-032116-094545 27216936

[6] Xu J, Jarocha LE, Zollitsch T et al. Nature 2021; 594: 535–40. 10.1038/s41586-021-03618-9 34163056

[7] Leberecht B, Wong SY, Satish B et al. Proc Natl Acad Sci USA 2023; 120: 2301153120. 10.1073/pnas.2301153120

[8] Bradlaugh AA, Fedele G, Munro AL et al. Nature 2023; 615: 111–16. 10.1038/s41586-023-05735-z 36813962 PMC9977682

[9] Ikeya N, Woodward JR. Proc Natl Acad Sci USA 2021; 118: e2018043118. 10.1073/pnas.2018043118 33397812 PMC7826401

[10] Pooam M, El-Esawi M, Aguida B et al. J Plant Biochem Biot 2020; 29: 636–51. 10.1007/s13562-020-00620-6

[11] Buchachenko AL, Bukhvostov AA, Ermakov KV et al. Prog Biophys Mol Biol 2020; 155: 1–19. 10.1016/j.pbiomolbio.2020.02.007 32224188

[12] Zadeh-Haghighi H, Simon C. J R Soc Interface 2022; 19: 20220325. 10.1098/rsif.2022.0325 35919980 PMC9346374

[13] Kim Y, Bertagna F, D'Souza EM et al. Quantum Rep 2021; 3: 1–48.

[14] Hore PJ . eLife 2019; 8: e44179. 10.7554/eLife.44179 30801245 PMC6417859

[15] Eckvahl HJ, Tcyrulnikov NA, Chiesa A et al. Science 2023; 382: 197–201. 10.1126/science.adj5328 37824648

