# Supplementary material for: Spin chemistry in living systems
Source: Natl Sci Rev. 2024 Mar 28;11(9):nwae126. doi: 10.1093/nsr/nwae126 (PMC11321246; doi:10.1093/nsr/nwae126)
Supplement: nwae126_Supplemental_File [file nwae126_supplemental_file.pdf]

Spin chemistry in living systems

P. J. Hore

Department of Chemistry, Oxford University, UK
Email: peter.hore@chem.ox.ac.uk

Supplementary Material

Could my magnetic field effect be due to the radical pair mechanism?

The literature on biological magnetic field effects has been extensively reviewed over the last 7 years [1-12]. That so many of these reports have been attributed to the RPM seems to reflect the scarcity of plausible alternatives. Attractive though it is to have a theoretical framework for interpreting data and designing experiments, the evidence for the involvement of radical pairs in some of these studies looks pretty thin. Links to radical pair theory are generally more convincing if the experimental observations conform to more than one of the known features of the mechanism. The following is a selection of the general characteristics of radical pairs which may be useful to anyone wishing to make a stronger case for assigning observed magnetic field effects to the RPM. Not all items in the list will be relevant or experimentally feasible for every suspected radical pair reaction. Some reactions may show none of these features, for example if the radicals are too strongly coupled or spin-relax too rapidly or are too short-lived. The list is not intended to be comprehensive. There are exceptions to most of the entries. Illustrative literature references are included.

- The most common manifestation of RPM effects is that reaction rates and/or product yields are altered by the application of static, radiofrequency and/or microwave magnetic fields [13-17]. The intensities and frequencies that are most likely to produce a significant change depend on many factors, including the identities of the radicals and their internal magnetic interactions, reaction rates, molecular motions, and so on [5].
- Very few convincing RPM effects have been reported for *chemical* systems subject to magnetic fields weaker than ~100 μT [18, 19]. This is testimony to how insensitive radical pairs are to very weak magnetic fields [20].
- Reaction rates and/or product yields may also be affected by isotopic substitution, provided the nuclides have sufficiently different hyperfine interactions [4, 21-23]. Other things being equal, the largest effects are expected when a non-magnetic nuclide is replaced by a magnetic one, or vice versa. Care needs to be taken that the isotopologues are pure and do not contain different concentrations of paramagnetic impurities which may induce fast spin relaxation.
- For static magnetic fields up to ~100 mT, the yields of radical pair reactions often show a characteristic sigmoidal dependence on the strength of the field. If the radicals are long-lived and slowly relaxing, the response may be biphasic as a result of the Low Field Effect [24]. The half-field parameter, $B_{1/2}$, is normally comparable to the effective hyperfine interaction of the two radicals [25]. Larger values of $B_{1/2}$ are expected if the radicals undergo rapid spin relaxation [26]. See Figure S1 for details.
- Applied magnetic fields and magnetic isotope substitution can increase or decrease product yields depending on the initial spin state of the radical pair (singlet or triplet) and whether the product is formed from the singlet or the triplet state of the radical pair or both. See Figure S1 for details.

- If the radicals are not randomly oriented, the effect of a static magnetic field may depend on the direction of the field (as a result of anisotropic hyperfine and/or dipolar interactions) [17-19].
- Exact reversal of the field direction should have no effect whether the radicals are oriented or not.
- Assignment of magnetic field/magnetic isotope effects to the RPM is much more convincing if the magnetically sensitive radicals can be identified, e.g. by optical or EPR spectroscopy. EPR detection is particularly convincing and informative if the radicals are spin-polarised in accordance with their structural, magnetic, and chemical properties [27, 28].
- Nuclear spin hyperpolarisation of the reaction products, observed by NMR, provides strong evidence for a radical pair precursor [29, 30]. This can be especially valuable when the radicals are too short-lived for EPR detection.
- For static fields much stronger than the hyperfine interactions, there should be a resonant radiofrequency/microwave field effect at the electron Larmor frequency (28 MHz per mT). This resonance should be strongest when the static and time-dependent fields are perpendicular and weakest when they are parallel. This is the nearest thing to a diagnostic test for the RPM [31].
- For weak static fields, the maximum radiofrequency that should have a resonant effect on a radical pair is determined by the separation of the highest and lowest energy levels of the hyperfine spin Hamiltonian [32-34].
- Microwave field effects at frequencies above ~10 GHz are normally only expected for static fields in excess of ~350 mT.
- If the radical pairs are formed photochemically, the magnetic field effects should be consistent with the wavelength-dependence of the absorption spectrum of the precursor chromophore.
- If the radical pairs are formed by the random encounter of independently formed radicals, the phase of the magnetic field effect will depend on the chemistry. See Figure S1 for details.
- The effects of weak magnetic fields are strongly attenuated if the exchange and/or dipolar interactions of the two electron spins exceed the electron Zeeman interaction. In the case of dipolar coupling, this means that the radicals should be more than ~3 nm apart for a 100 μT magnetic field to have strong effect [35].
- The radicals $O_2^{\bullet-}$, $OH^{\bullet}$, and $NO^{\bullet}$ have orbitally degenerate ground states and therefore strong spin-orbit coupling and, normally, very fast spin relaxation (nanoseconds). As a result, extremely small magnetic field effects can be expected [36, 37]. Organic radicals usually have longer spin-relaxation times (rarely longer than 1 μs).
- Effects of extremely low frequency (ELF) magnetic fields (e.g. 50/60 Hz) at the levels to which humans are routinely exposed (~1 μT) are, in all probability, no more than 10 parts per million [20].

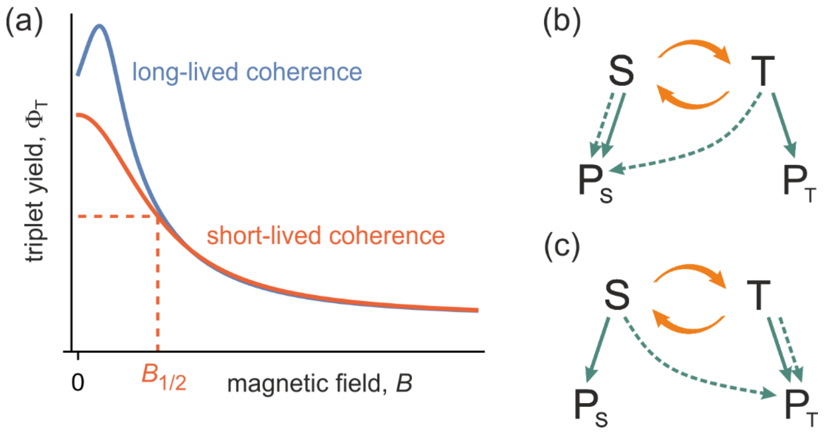

Figure S1. Radical pair magnetic field effects and reaction schemes. (a) Typical dependence of the yield (Φ_T) of the triplet product (P_T) of a singlet-born radical pair on the strength of a weak static magnetic field (B). The two lines show the behaviour expected for radical pairs with short-lived (red) and long-lived (blue) spin coherence. For the red line, $B_{1/2}$ is the magnetic field at which Φ_T is equal to the mean of its value at zero field and its limiting value at high field. (b) and (c) Radical pair reaction schemes. S and T are the singlet and triplet states of the radical pair. The curved orange arrows indicate the coherent interconversion of the S and T states by hyperfine and Zeeman interactions. S and T are able to react spin-selectively to form singlet and triplet products, P_S and P_T, respectively (solid green arrows). Only one of the two reactions needs to be spin-selective: either P_S (b) or P_T (c) can additionally be formed by non-spin-selective reactions (dashed arrows). Examples of non-spin selective processes include reactions that involve only one of the radicals, e.g. protonation/deprotonation via an external proton donor/acceptor [38-42]. Scheme (c) is also appropriate if both components of the pair diffuse apart to form free radicals while only the singlet pairs recombine [43, 44].

Notes

- Whether the radical pair is singlet-born or triplet-born is determined by whether it is formed spin-selectively from a singlet or a triplet precursor, respectively.
- Both traces in (a) should be inverted to obtain the yield of the *singlet* product, $\Phi_S = 1 - \Phi_T$.
- Both traces in (a) should be inverted for a *triplet-born* radical pair.
- The value of $B_{1/2}$ is typically similar to the effective hyperfine interaction of the radical pair (typically a few millitesla). Larger values are often the result of fast spin relaxation.
- F-pairs are radical pairs formed by the random encounter of independently formed radicals with initially uncorrelated electron spins. F-pairs behave qualitatively like triplet-born pairs if the singlet pair is shorter lived than the triplet and like singlet-born pairs if the opposite is true.
- The shape of the red line in (a) arises from the inhibition of hyperfine-induced S-$T_{\pm 1}$ interconversion by the Zeeman interaction.
- The initial rise of the blue line in (a) is the Low Field Effect which arises from the removal of zero-field energy-level degeneracies.
- Low Field Effects occur for magnetic fields in which the Larmor period, $(\gamma_e B / 2\pi)^{-1}$, is longer than the spin-coherence lifetime.

Cryptochrome-related magnetic field effects: publications and citations

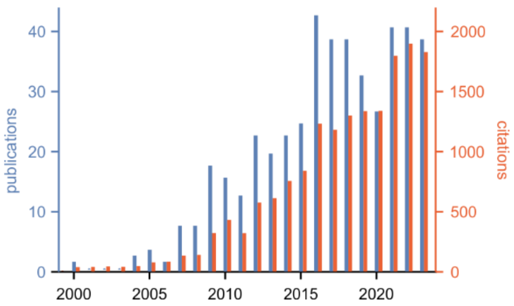

Figure S2. **Cryptochrome-related magnetic field effects**. Results of a Web of Science search for the topic "cryptochrome* and magnet*" (21 January 2024). 441 publications were found, with a total of 15,610 citations including 9,031 without self-citations. *h*-index = 63. The paper that started it all off is "A model for photoreceptor-based magnetoreception in birds", T. Ritz, S. Adem and K. Schulten, *Biophys. J.* 78 (2000) 707-718.

Reaction schemes

Figures S3-S6 show reaction schemes for some of the radical pair processes mentioned in the main text. In all four figures, red and blue colours denote, respectively, singlets (S) and triplets (T) and their spin-selective reactions. The curved red/blue arrows represent the coherent interconversion of the S and T states of the radical pairs.

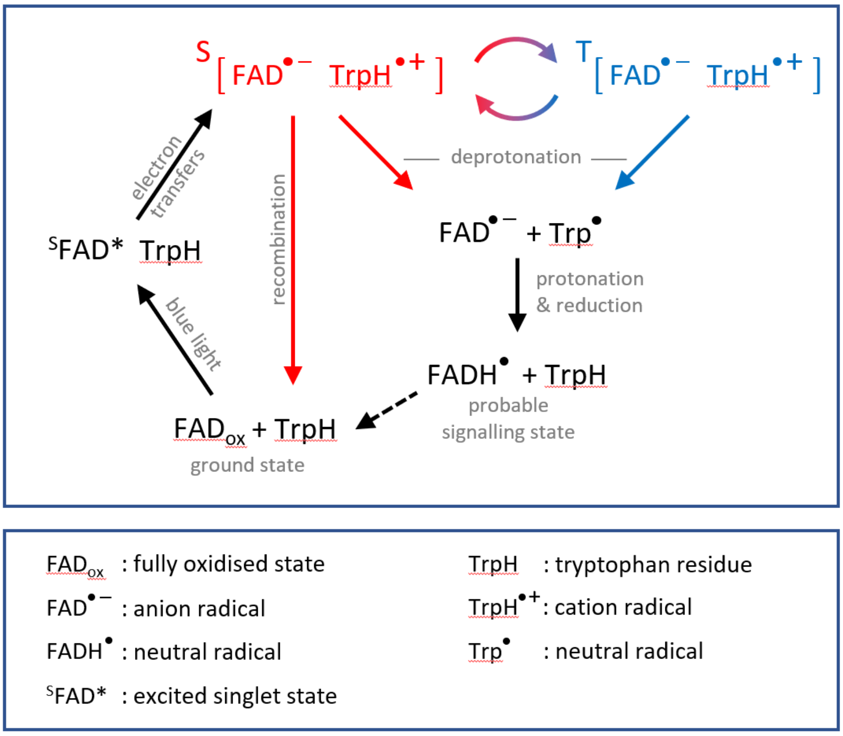

Figure S3. Cryptochrome photo-reaction scheme [17, 42]. FAD and TrpH are the flavin adenine dinucleotide cofactor and the terminal tryptophan of the electron transfer chain. Magnetic field effects arise from competition between spin-allowed recombination of the singlet radical pair and deprotonation of $TrpH^{\bullet+}$. Subsequent reactions include protonation of $FAD^{\bullet-}$ and reduction of $Trp^{\bullet}$. The intermediate electron transfer steps between $^{S}FAD^*$ and $[FAD^{\bullet-}\ TrpH^{\bullet+}]$ are not shown.

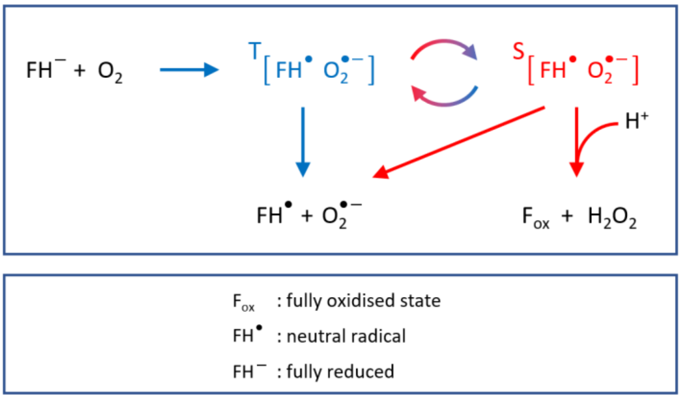

Figure S4. Reaction scheme for the production of superoxide $O_2^{\bullet-}$ and hydrogen peroxide (H_2O_2) from O_2 and a fully reduced flavin [45]. The formation of F_{ox} and H_2O_2 from the singlet radical pair could occur via a flavin C(4a) hydroperoxide intermediate or a second one-electron transfer. The extremely fast spin relaxation of $O_2^{\bullet-}$ (see main text) makes it highly unlikely that a $[\,FH^{\bullet}\ O_2^{\bullet-}\,]$ radical pair could be a source of magnetic field effects.

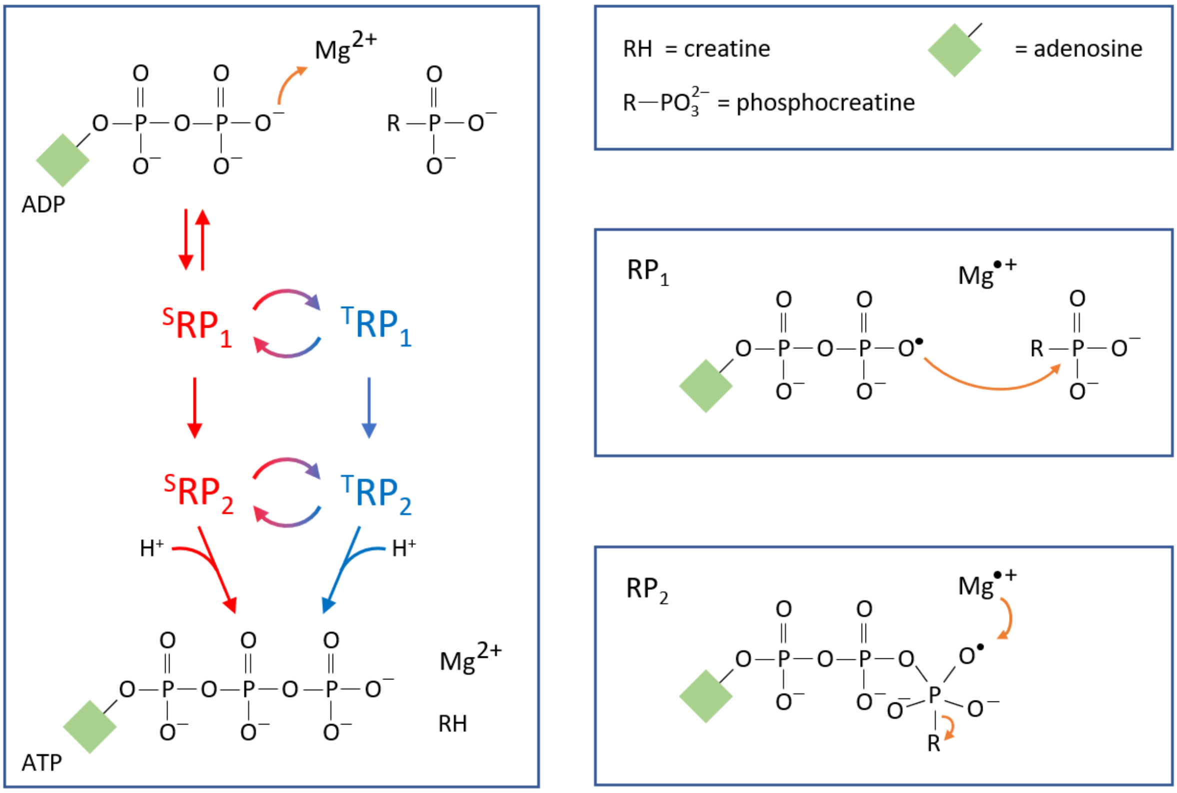

Figure S5. Proposed reaction scheme for magnesium-dependent conversion of ADP to ATP by creatine kinase [23, 46, 47].

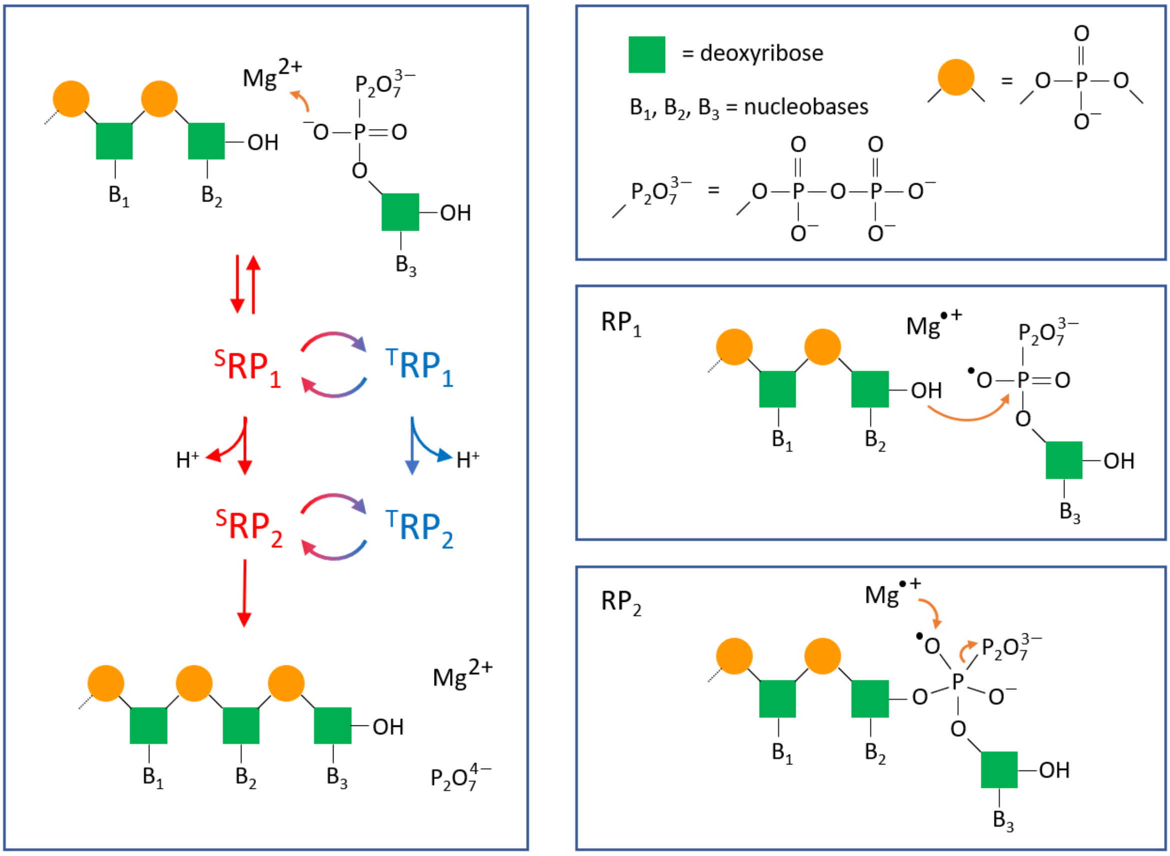

Figure S6. Proposed reaction scheme for magnesium-dependent DNA replication [48, 49].

REFERENCES

1. Binhi VN, Prato FS. Biological effects of the hypomagnetic field: an analytical review of experiments and theories. *PLoS ONE*. 2017; **12**: e0179340.
2. Zhang XL, Yarema K, Xu A. *Biological effects of static magnetic fields*. Singapore: Springer, 2017.
3. Pooam M, El-Esawi M, Aguida B, Ahmad M. Cryptochrome and quantum biology: new insights for plant science and crop improvement. *J Plant Biochem Biot*. 2020; **29**: 636-651
4. Buchachenko AL, Bukhvostov AA, Ermakov KV, Kuznetsov DA. A specific role of magnetic isotopes in biological and ecological systems. Physics and biophysics beyond. *Prog Biophys Mol Biol*. 2020; **155**: 1-19
5. Kim Y, Bertagna F, D'Souza EM *et al.* Quantum biology: An update and perspective. *Quantum Rep*. 2021; **3**: 1-48
6. Binhi VN, Rubin AB. Theoretical concepts in magnetobiology after 40 years of research. *Cells*. 2022; **11**: 274
7. Zadeh-Haghighi H, Simon C. Magnetic field effects in biology from the perspective of the radical pair mechanism. *J R Soc Interface*. 2022; **19**: 20220325
8. Ueno S, Shigemitsu T. *Bioelectromagnetism. History, foundations and applications*. Boca Raton: CRC Press, 2022.
9. Koltover VK. Magnetic isotope effects and nuclear spin catalysis in living cells and biomolecular motors: recent advances and future outlooks. *Biophy Rev*. 2023; **15**: 999–1006
10. Sarimov RM, Serov DA, Gudkov SV. Hypomagnetic conditions and their biological action. *Biology*. 2023; **12**: 1513
11. Sarimov RM, Serov DA, Gudkov SV. Biological effects of magnetic storms and ELF magnetic fields. *Biology*. 2023; **12**: 1506
12. Buchachenko AL. *Magnetic effects across biochemistry, molecular biology and environmental chemistry*. London: Academic Press, 2024.
13. Steiner UE, Ulrich T. Magnetic field effects in chemical kinetics and related phenomena. *Chem Rev*. 1989; **89**: 51-147
14. Woodward JR. Radical pairs in solution. *Prog React Kinet Mech*. 2002; **27**: 165-207
15. Rodgers CT. Magnetic field effects in chemical systems. *Pure Appl Chem*. 2009; **81**: 19-43
16. Jones AR. Magnetic field effects in proteins. *Molec Phys*. 2016; **114**: 1691-1702
17. Hore PJ, Mouritsen H. The radical pair mechanism of magnetoreception. *Annu Rev Biophys*. 2016; **45**: 299-344
18. Maeda K, Henbest KB, Cintolesi F *et al.* Chemical compass model of avian magnetoreception. *Nature*. 2008; **453**: 387-390

19. Kerpal C, Richert S, Storey JG *et al.* Chemical compass behaviour at microtesla magnetic fields strengthens the radical pair hypothesis of avian magnetoreception. *Nat Comm*. 2019; **10**: 3707
20. Hore PJ. Upper bound on the biological effects of 50/60 Hz magnetic fields mediated by radical pairs. *eLife*. 2019; **8**: e44179
21. Salikhov KM. *Magnetic isotope effect in radical reactions*. Vienna: Springer-Verlag, 1996.
22. Buchachenko AL. *Magnetic isotope effect in chemistry and biochemistry*. New York: Nova Science Publishers, 2009.
23. Crotty D, Silkstone G, Poddar S *et al.* Re-examination of magnetic isotope and magnetic field effects on adenosine triphosphate production by creatine kinase. *Proc Natl Acad Sci USA*. 2011; **109**: 1437-1442
24. Timmel CR, Till U, Brocklehurst B *et al.* Effects of weak magnetic fields on free radical recombination reactions. *Molec Phys*. 1998; **95**: 71-89
25. Wong SY, Benjamin P, Hore PJ. Magnetic field effects on radical pair reactions: estimation of $B_{1/2}$ for flavin-tryptophan radical pairs in cryptochromes. *Phys Chem Chem Phys*. 2023; **25**: 975-982
26. Golesworthy M, Zollitsch T, Luo J *et al.* Singlet-triplet dephasing in radical pairs in avian cryptochromes leads to time-dependent magnetic field effects. *J Chem Phys*. 2023; **159**: 105102
27. Forbes MDE, Jarocha LE, Sim S, Tarasov VF. Time-resolved electron paramagnetic resonance spectroscopy: history, technique, and application to supramolecular and macromolecular chemistry. *Adv Phys Org Chem*. 2013; **47**: 1-83
28. Biskup T. Time-resolved EPR of radical pair intermediates in cryptochromes. *Mol Phys*. 2013; **111**: 3698-3703
29. Matysik J, Ding Y, Kim Y *et al.* Photo-CIDNP in solid state. *Appl Magn Reson*. 2022; **53**: 521-537
30. Lee JH, Okuno Y, Cavagnero S. Sensitivity enhancement in solution NMR: Emerging ideas and new frontiers. *J Magn Reson*. 2014; **241**: 18-31
31. Henbest KB, Kukura P, Rodgers CT *et al.* Radio frequency magnetic field effects on a radical recombination reaction: a diagnostic test for the radical pair mechanism. *J Amer Chem Soc*. 2004; **126**: 8102-8103
32. Hiscock HG, Mouritsen H, Manolopoulos DE, Hore PJ. Disruption of magnetic compass orientation in migratory birds by radiofrequency electromagnetic fields. *Biophys J*. 2017; **113**: 1475-1484
33. Leberecht B, Kobylkov D, Karwinkel T *et al.* Broadband 75-85 MHz radiofrequency fields disrupt magnetic compass orientation in night-migratory songbirds consistent with a flavin-based radical pair magnetoreceptor. *J Comp Physiol A*. 2022; **208**: 97-106

34. Leberecht B, Wong SY, Satish B *et al.* Upper bound for broadband radiofrequency field disruption of magnetic compass orientation in night-migratory songbirds. *Proc Natl Acad Sci USA*. 2023; **120**: 2301153120
35. Babcock NS, Kattnig DR. Electron–electron dipolar interaction poses a challenge to the radical pair mechanism of magnetoreception. *J Phys Chem Lett*. 2020; **11**: 2414-2421
36. Karogodina TY, Sergeeva SV, Stass DV. Magnetic field effect in the reaction of recombination of nitric oxide and superoxide anion. *Appl Magn Reson*. 2009; **36**: 195-208
37. Karogodina TY, Dranov IG, Sergeeva SV *et al.* Kinetic magnetic-field effect involving the small biologically relevant inorganic radicals nitric oxide and superoxide. *Chem Phys Chem*. 2011; **12**: 1714-1728
38. Maeda K, Robinson AJ, Henbest KB *et al.* Magnetically sensitive light-induced reactions in cryptochrome are consistent with its proposed role as a magnetoreceptor. *Proc Natl Acad Sci USA*. 2012; **109**: 4774-4779
39. Kattnig DR, Evans EW, Déjean V *et al.* Chemical amplification of magnetic field effects relevant to avian magnetoreception. *Nat Chem*. 2016; **8**: 384-391
40. Evans EW, Kattnig DR, Henbest K *et al.* Sub-millitesla magnetic field effects on the recombination reaction of flavin and ascorbic acid radicals. *J Chem Phys*. 2016; **145**: 085101
41. Sheppard DMW, Li J, Henbest KB *et al.* Millitesla magnetic field effects on the photocycle of *Drosophila melanogaster* cryptochrome. *Sci Rep*. 2017; **7**: 42228
42. Xu J, Jarocha LE, Zollitsch T *et al.* Magnetic sensitivity of cryptochrome 4 from a migratory songbird. *Nature*. 2021; **594**: 535-540
43. Henbest KB, Athanassiades E, Maeda K *et al.* Photoionization of TMPD in DMSO solution: mechanism and magnetic field effects. *Mol Phys*. 2006; **104**: 1789-1794
44. Rodgers CT, Norman SA, Henbest KB *et al.* Determination of radical re-encounter probability distributions from magnetic field effects on reaction yields. *J Amer Chem Soc*. 2007; **129**: 6746-6755
45. Massey V. Activation of molecular oxygen by flavins and flavoproteins. *J Biol Chem*. 1994; **269**: 22459-22462
46. Buchachenko AL, Kouznetsov DA, Orlova MA, Markarian AA. Magnetic isotope effect of magnesium in phosphoglycerate kinase phosphorylation. *Proc Natl Acad Sci USA*. 2005; **102**: 10793-10796
47. Buchachenko AL, Kouznetsov DA, Breslavskaya NN, Orlova MA. Magnesium isotope effects in enzymatic phosphorylation. *J Phys Chem B*. 2008; **112**: 2548-2556
48. Stovbun SV, Zlenko DV, Bukhvostov AA *et al.* Magnetic field and nuclear spin influence on the DNA synthesis rate. *Sci Rep*. 2023; **13**: 465

49. Buchachenko AL, Kuznetsov DA. Genes and cancer under magnetic control. *Russ J Phys Chem B*. 2021; **15**: 1-11
